# Oxygen therapy in patients with retinal artery occlusion: A meta-analysis

**DOI:** 10.1371/journal.pone.0202154

**Published:** 2018-08-29

**Authors:** Xiaodong Wu, Shuangshuang Chen, Shizun Li, Ji Zhang, Di Luan, Shoucai Zhao, Zhaohu Chu, Yang Xu

**Affiliations:** 1 Department of Neurology, Wannan Medical College First Affiliated Hospital, Yijishan Hospital, Wuhu, China; 2 Central Laboratory, Wannan Medical College First Affiliated Hospital, Yijishan Hospital, Wuhu, China; Edith Wolfson Medical Center, ISRAEL

## Abstract

**Background:**

Oxygen therapy has been widely used for RAO (retinal artery occlusion) patients; however, inconsistent results have been reported.

**Methods:**

PubMed, Web of Science, EMBASE, Medline (OvidSP), Cochrane, China National Knowledge Infrastructure (CNKI), and Wanfang Database were examined. The primary endpoint was visual acuity (VA), and RevMan software 5.3 was used to statistically analyze the outcomes.

**Results:**

Seven randomized controlled trials (RCTs) met the inclusion criteria. Patients who received oxygen therapy exhibited probability of visual improvement about 5.61 times compared with the control group who did not receive oxygen therapy (OR = 5.61; 95% CI, 3.60–8.73; *p* < 0.01). No statistically significant difference was observed between oxygen inhalation methods (Chi^2^ = 0.18, df = 1, *p* = 0.67), combined therapy (Chi^2^ = 0.21, df = 1, *p* = 0.64), or RAO type (Chi^2^ = 0.06, df = 1, *p* = 0.81). Conversely, 100% oxygen (Chi^2^ = 4.55, df = 1, *p* < 0.05) and hyperbaric oxygen (Chi^2^ = 4.55, df = 1, *p* < 0.05) significantly improved VA in RAO patients. Better effect was showed in period within 3 months (Chi^2^ = 5.76, df = 1, *p* < 0.05). The most effective treatment length was over 9 hours (Chi^2^ = 6.58, df = 1, *p* < 0.05).

**Conclusion:**

Oxygen therapy demonstrated beneficial effects in improving VA in RAO patients, particularly when patients were treated with 100% hyperbaric oxygen and for over 9 hours.

## Introduction

Retinal artery occlusion (RAO) is a serious event, which causes restriction in eyesight [1]. Central retinal artery occlusion (CRAO) was first reported in 1859 [2]. The ophthalmic artery originates from the internal carotid artery, and the central retinal artery (CRA) is a small important branch of the ophthalmic artery. The blood supply of the inner layer of the retina comes from the CRA and its branches; occlusion of the branch leads to a branch retinal artery occlusion (BRAO) [3]. The aetiology of RAO includes thrombosis, embolus, arteritis, vasospasm [4]. Clinically, the consequences of this vascular accident are dramatic, and delayed treatment may cause blindness; RAO is more common in hypertensive arteriosclerosis patients and occurs occasionally patients with endocarditis [5, 6]. Visual loss is a major symptom in CRAO, while limited vision field has been described in BRAO. Despite great developments in diagnostic, surgical and medical ophthalmology fields within recent years, retinal artery occlusion (RAO) remains a disease without approved therapy. Retinal cells exhibited the highest oxygen consumption in organs, which makes the retina extremely susceptible to ischaemia [7]. The inner retinal layers are normally supported by retinal circulation and typically lose viability, leading to vision loss. However, providing sufficient amounts of oxygen may improve visual acuity [8]. Traditional treatments for RAO include ocular massage, haemodilution, anterior chamber paracentesis, intravenous acetazolamide, oxygen therapy, transluminal Nd:YAG laser, intra-arterial thrombolytic therapy, intravenous fibrinolytic therapy and among all conventional conservative methods, most have not shown significant improvement [9–16]. Intravenous fibrinolytic therapy may also induce serious haemorrhagic events, and the time of symptom onset is critical to be safe and effective [17]. In terms of oxygen therapy, the outcome remains inconsistent [18–20]. Because there are few randomized controlled trials (RCTs) and many case reports in the literature, there has been no meta-analysis on oxygen therapy in RAO patients. Thus, we report this meta-analysis to provide a treatment reference for the use of oxygen therapy in RAO patients.

## Materials and methods

### Search strategy

We searched the literature in PubMed, Web of Science, EMBASE, Medline (OvidSP), Cochrane, China National Knowledge Infrastructure (CNKI), and Wanfang Database for articles published between the inception of the database to May 16, 2018. No language criteria were applied, and the following keywords were used: “normobaric oxygen” or “hyperbaric oxygen” or “oxygen” AND “retinal artery occlusion” OR “RAO”.

### Inclusion and exclusion criteria

The inclusion criteria were as follows: A) research subjects should be patients diagnosed with RAO; B) all studies must be RCTs; C) the intervention group received oxygen therapy; and D) the best corrected visual acuity (VA) was compared between the oxygen therapy group and non-oxygen therapy group. The exclusion criteria were as follows: A) animal models; B) not related to the disease of RAO; C) not an intervention of oxygen therapy; and D) VA was not an endpoint.

### Data extraction and risk of bias in included studies

Two investigators (Xiaodong Wu and Shuangshaung Chen) independently selected studies according to the abovementioned criteria. The following information was reported: first author’s name, year of publication, time from onset, oxygen pressure, oxygen inhalation method, length of treatment, combined therapy. Each risk of bias item was independently evaluated with the risk of bias software produced by the Cochrane Collaboration [21]. Any ambiguity or disagreement was resolved by a third investigator (Yang Xu).

### Statistical analysis

Forest plots and funnel plots were generated to analyse the outcomes, and publication bias was detected using the Cochrane Collaboration’s RevMan 5.3 software. For each study, ORs and corresponding 95% CIs were estimated. A fixed-effect model was used for this meta-analysis to reduce errors for more accurate results. I^2^ reflects the heterogeneity of the proportion of total variation in the amount of the effect. According to I^2^, the degree of heterogeneity can be divided into three levels: 0 indicates no heterogeneity; 0–50% indicates low heterogeneity; 50%-75% indicates a moderate level of heterogeneity; 75%-100% indicates a high level of heterogeneity. Subgroup analysis was produced to search for the source if significant heterogeneity occurs. Chi-square test for metrological data analysis applied to subgroup analysis, p < 0.05 was considered a significant difference in the compared groups.

## Results

### Study inclusion and study characteristics

**Fig 1:**
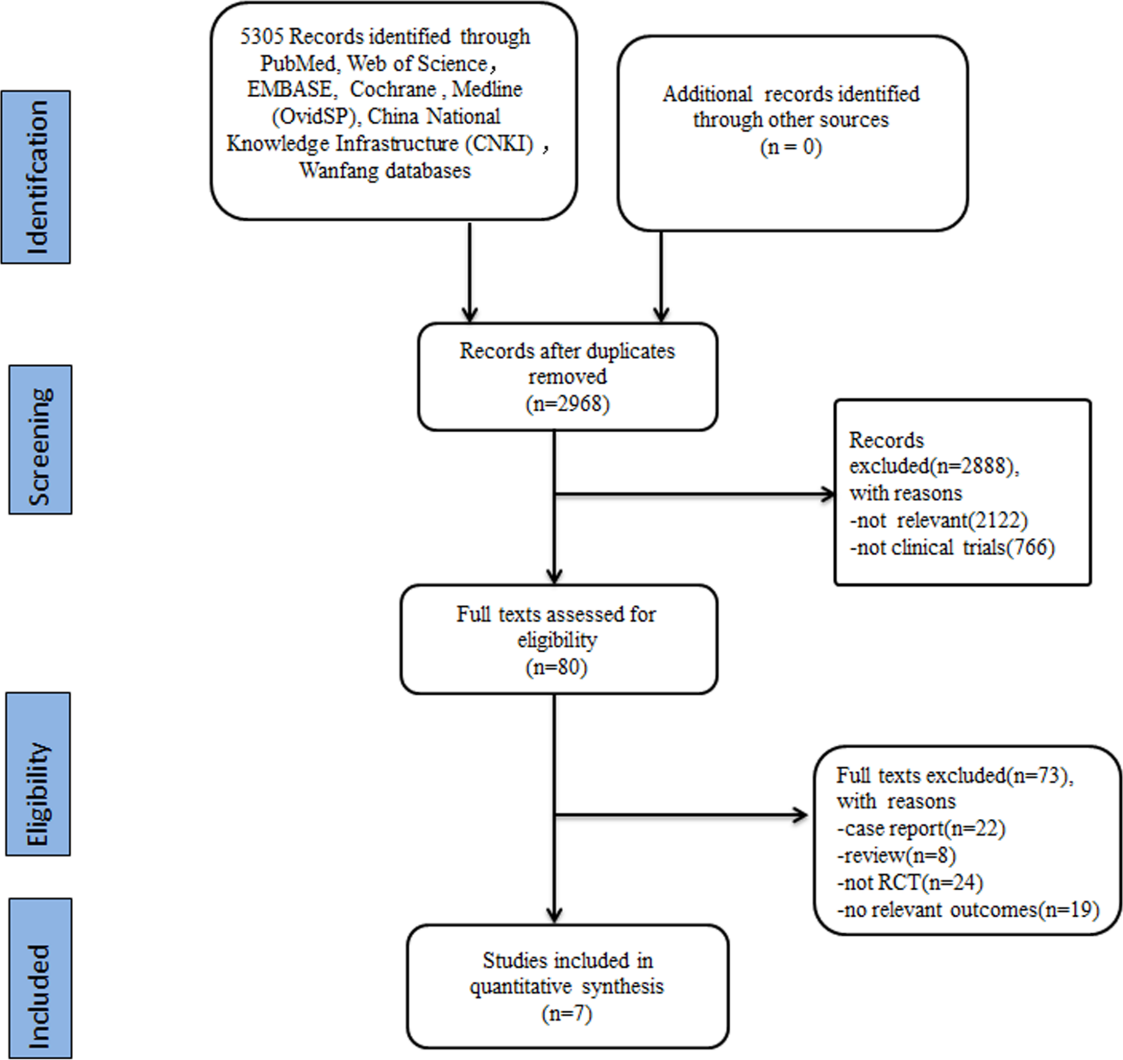
Flow diagram of studies retrieval and screening.

(Fig 1) shows a schematic of the study design. The search strategies initially identified 5305 papers. After removing duplications, 2968 articles were found. Next, 2888 papers were excluded for the following specific reasons: not relevant (n = 2122), no clinical trials (n = 766). Among the remaining 80 articles, 73 articles were removed due to being case reports (n = 22), being reviews (n = 8), not being an RCT (n = 24), or not reporting relevant outcomes (n = 19). Seven studies were finally included in this meta-analysis [22–28].Three studies were published in English [23–25], three studies were published in Chinese [26–28], and one study was published in German [22]. (Table 1) showed the main characteristics of the 7 studies.

**Table 1:** Statistical characteristics of included studies.

Author	Language	Year	Patient	Time from onset	Oxygen treatment	Oxygen inhalation method	Total length of treatment	Outcome	Patients	Recovery, No.(%)	Time point of vision evaluation	Combined Treatment
Neal H. Atebara	English	1995	CRAO	≤24h	1ATA, 95%O2 + 5%CO2	Face mask	4 hours	VA	40	4, 10%	4months-5years	Anterior chamber paracentesis
Zhang	Chinese	2000	CRAO	4h-5d	2.4ATA, 100%	Face mask	16.5h	VA	32	28, 87.5%	1.5months	VitB1,B12

S Aisenbrey	German	2000	CRAO + BRAO	4-12h	2.4ATA, N	N/A	16.5h	VA	18	12, 66.7%	3months	Ocular massage acetazolamide
Beiran I	English	2001	CRAO + BRAO	≤8h	2.8 ATA, 100%	N/A	9h	VA	35	29, 82.9%	Discharge	Ocular massage, acetazolamide, retrobulbar block,
												paracentesis
He	Chinese	2009	CRAO	2h-5d	2.4ATA,	Face mask	17h	VA	20	18, 90%	1month	TMP,VitB1

Johannes	English	2012	CRAO	≤12h	2.4ATA, 100%,	Face mask	7.5h	VA	51	30, 58.8%	Discharge	Haemodilution therapy
Wang	Chinese	2016	CRAO	0.5h-2d	2.0–2.5ATA, 100%	Face mask	24h	VA	55	45, 81.8%	10-12days	None

ATA: atmosphere absolute; TMP: tetramethylpyrazine.

### Overall efficacy

**Fig 2:**
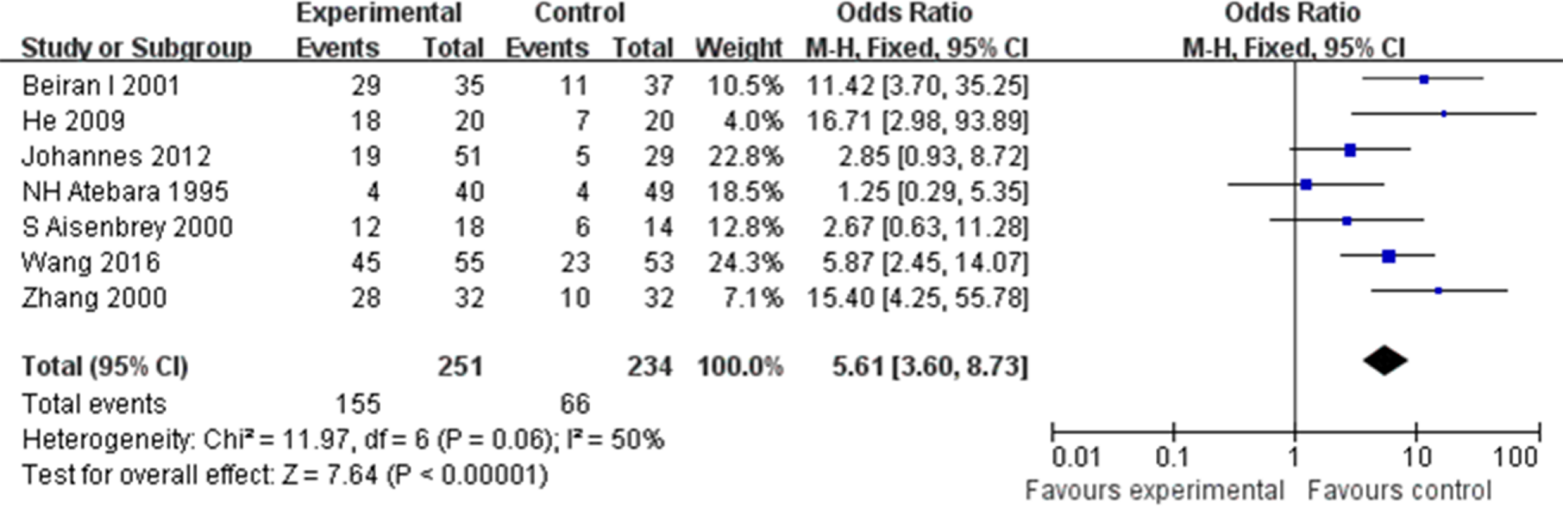
Forest plots for overall analyses: Oxygen therapy vs Control, outcome: Visual acuity.

Seven studies that include 251 patients described VA improvement treated with oxygen therapy. Oxygen therapy exhibited significant VA improvement in RAO patients compared with the non-oxygen therapy group (OR 5.61; 95% CI, 3.60–8.73) in a fixed-effect model, with heterogeneity (I^2^ = 50%, *p* = 0.06) (Fig 2). Patients who received oxygen therapy exhibited probability of visual improvement about 5.61 times compared with the control group who did not receive oxygen therapy.

### Prespecified sub-group analyses

**Fig 3:**
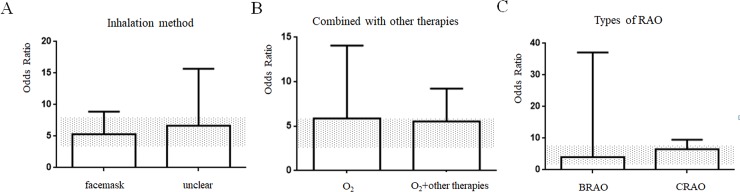
(A) Inhalation method, (B) Combined with other therapies, (C) Types of RAO. The 95% CI for the global estimate is presented as a grey stripe.

**Fig 4:**
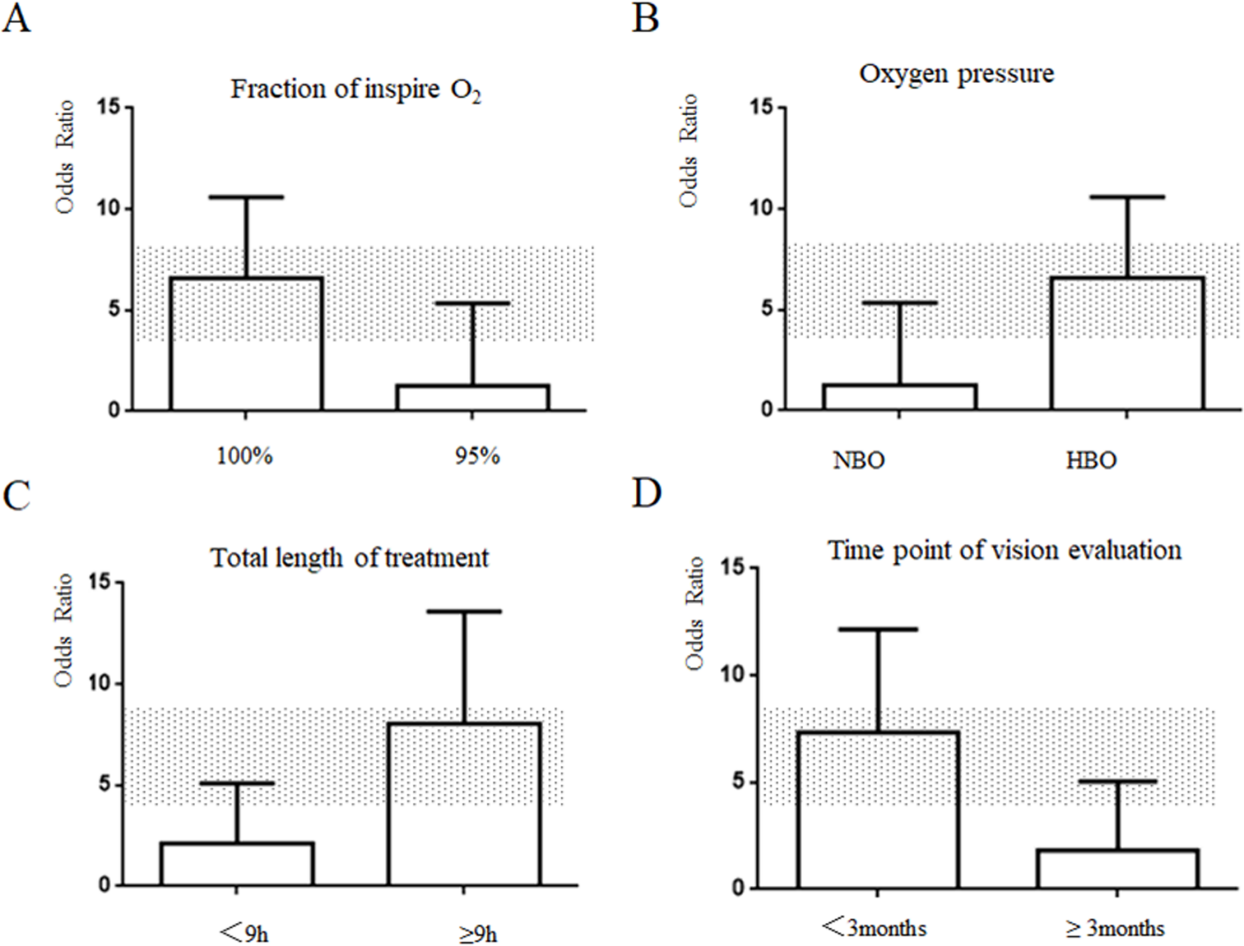
(A) Fraction of inspire O_2_, (B) Oxygen pressure, (C) Length of treatment. (D) Time point of vision evaluation. The 95% CI for the global estimate is presented as a grey stripe.

To exclude the effect of confounding factors, we divided the included studies into 6 subgroups according to oxygen inhalation method (mask or unclear way), treatment therapy (oxygen therapy only or combined with other therapies), type of RAO (BRAO or CRAO), fraction of inspiration O_2_ (FiO_2_) (100% or 95%), treatment length (≤ 9 h or > 9 h), pressure of oxygen (normobaric oxygen or hyperbaric oxygen). Five studies [23, 25–28] used a facemask providing oxygen compared with two studies [22, 24] providing oxygen in an unclear way. The inhalation method of using a facemask was not significantly different than using unclear methods (Chi^2^ = 0.18, df = 1, *p* = 0.67) (Fig 3A). There was no statistically significant difference observed between oxygen therapy alone and oxygen therapy combined with other therapies in the included literature (Chi^2^ = 0.21, df = 1, *p* = 0.64) (Fig 3B). Types of RAO (BRAO or CRAO) showed little difference on VA outcome (Chi^2^ = 0.06, df = 1, p = 0.81) (Fig 3C). Additionally, 100% oxygen was associated with a significant increase in VA improvement compared with methods using 95% oxygen (Chi^2^ = 4.55, df = 1, *p* < 0.05) (Fig 4A). A significant difference was observed between the normobaric oxygen (NBO) group and hyperbaric oxygen (HBO) group, demonstrating that oxygen pressure played an important role in VA improvement (Chi^2^ = 4.55, df = 1, *p* < 0.05) (Fig 4B). Over 9 hours of oxygen treatment exhibited a better effect compared with treatments shorter than 9 hours, and this trend was significant (Chi^2^ = 6.58, df = 1, *p* < 0.05) (Fig 4C). Better effect was showed after treatment in period within 3 months, it showed significant statistical difference (Chi^2^ = 5.76, df = 1, *p* < 0.05) (Fig 4D)

### Risk of bias in included studies

**Fig 5:**
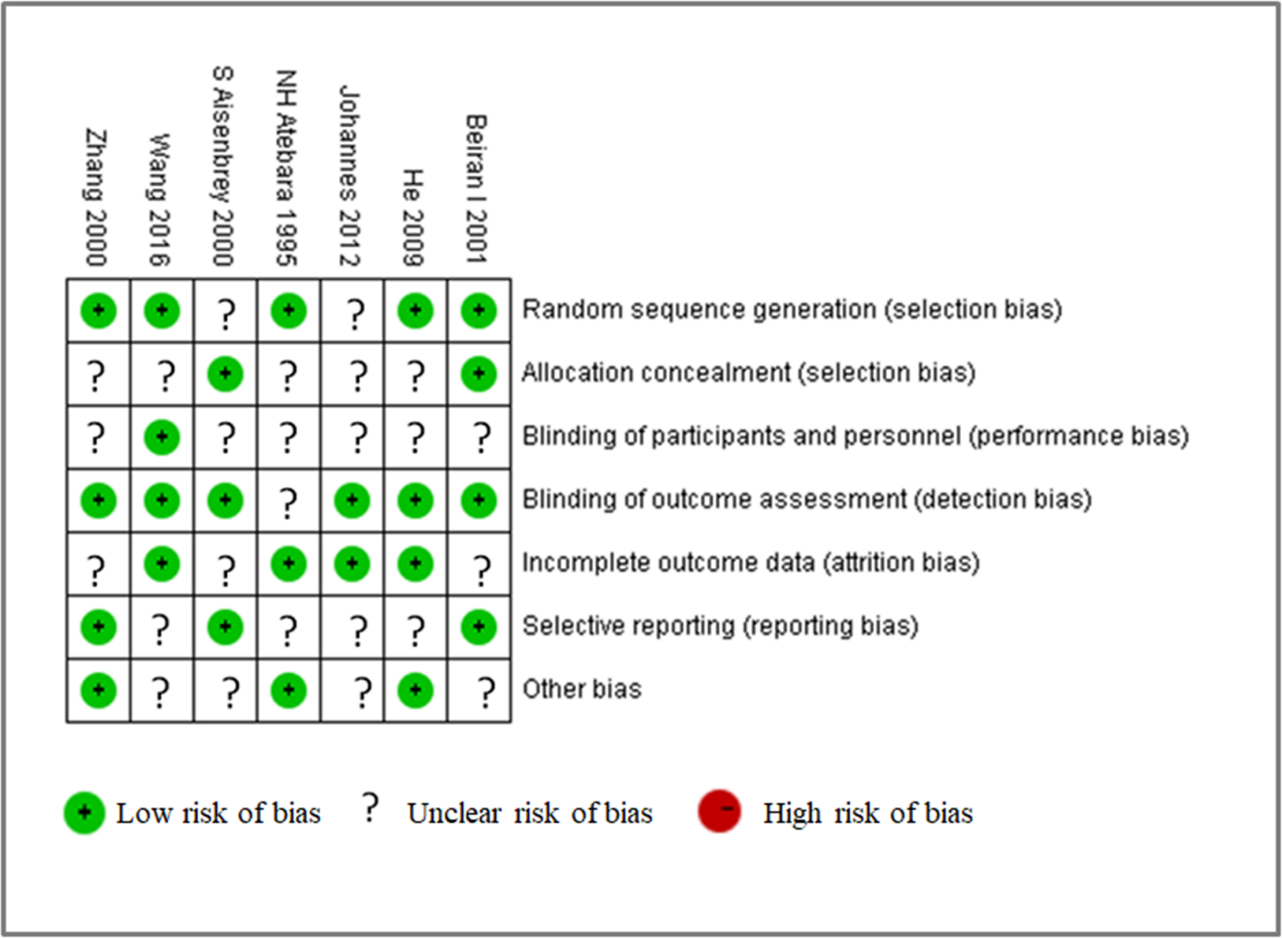
Risk of bias summary.

**Fig 6:**
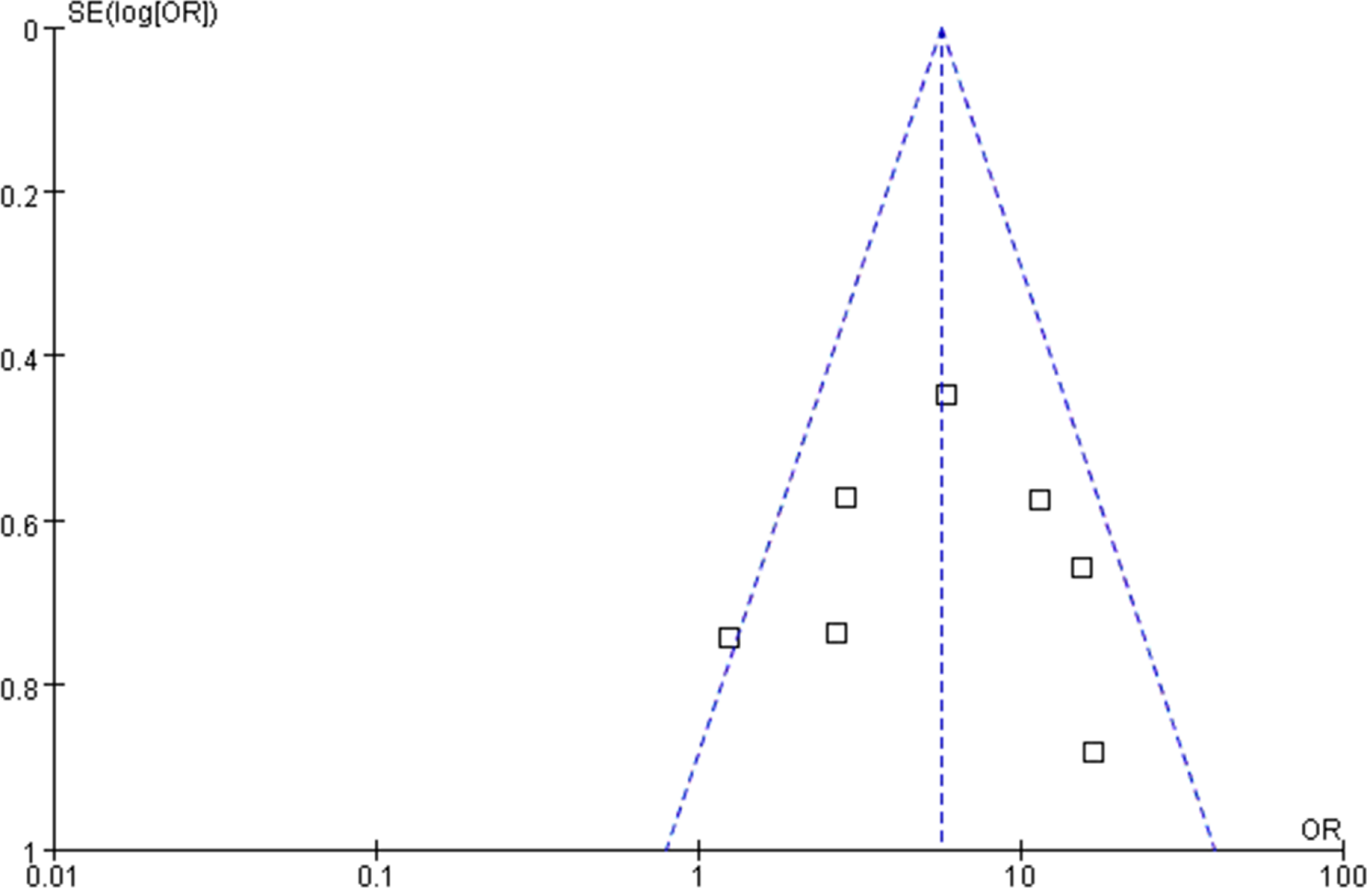
Funnel plot evaluating publication bias.

The quality of the 7 included studies was assessed using the Cochrane risk-of-bias tool, RevMan software 5.3 (Fig 5). Most studies showed low risk of bias. In terms of selection bias or detection bias, 86% of the included studies showed low risk, and 14% exhibited unclear risk. Moreover, three studies showed low risk and four studies showed unclear risks in reporting bias or other bias. Furthermore, 57% and 43% of the studies showed low and unclear risks of attrition bias, respectively. One study showed low risk of performance bias, and the other six studies showed an unclear risk of performance bias. All included studies showed the presence of publication bias (Fig 6).

## Discussion

In this meta-analysis, seven studies that met the inclusion criteria focused on the effectiveness of oxygen therapy in RAO patients. The studies showed that RAO patients treated with oxygen demonstrated improvement in VA compared with the non-oxygen therapy group. Based on these results, oxygen therapy is considered an effective method of treatment for RAO diseases. RAO exhibits similar vascular risk factors with stroke and is caused by various aetiologies. Hayreh found that marked improvement in visual acuity and visual field can occur without treatment and is determined by different factors [29]. Retinal tissue is not tolerant of hypoxia [30]. The inner retinal layers may receive sufficient oxygen through the diffusion of the choroidal circulation to sustain viability if increased FiO_2_ is supported. Normally, choroidal circulation supplies most of the oxygen to the retina. In the above analyses, no difference was observed in the method of oxygen inhalation, combined therapies, or types of RAO. Oxygen therapy plays a key role in these patients when combined with other therapies [22–26, 28].

It has been reported that HBO can be used to treat the disease and achieved good results [24, 31–35]. Oxygen therapy reduced the risk of retinal infarction by increasing tissue oxygen saturation [36]. A previous study showed that hyperoxic conditions can provide 100% of the oxygen demanded by the retina [37]. Hyperbaric oxygen rapidly increases blood oxygen tension and blood oxygen content, effectively improving the hypoxia status of retinal tissue and preventing retinal inner cells loss, thereby contributing to the recovery of reversible lesions. An animal experiment in a CRAO mice model showed that hyperbaric oxygen therapy diminished cell loss from 58% to 30%, which was related to increased survival of cells in the retinal inner layer [38]. In terms of visual field defect in BRAO, a study showed BRAO observed within one week from onset, inferior nasal accounted for 29%, more than central scotoma, superior sector and central inferior altitudinal defect. The natural history was central and peripheral visual field defect improved in 47% and 52%, respectively [39].

All the included 7 studies obtained individual-level data from 251 patients who received oxygen therapy with a particular focus on defining treatment length. The study showed that over 9 hours of treatment length increased the therapeutic effect. More than 9 hours of treatment are effective for restoring a patient’s vision. A case presentation revealed that a CRAO patient who was treated with hyperbaric oxygen therapy for a total of 13.5 hours gained marked visual acuity [40]. The time between onset and starting oxygen therapy is critical in RAO. There is a threshold of time beyond which the inner retinal cells can no longer recover from a hypoxic event, even if reperfusion occurs [37]. In the included 7 articles, the timing of treatment from onset cannot be statistically analyzed because the patient selection criteria in all RCTs required a time window within a specific time point. Nevertheless, a study showed that five patient cases with CRAO lasting over one and one-half hours demonstrated restored obvious improvement in visual acuity [41]. This finding further proves our viewpoint described above. Short-term effect showed higher probability of visual improvement than long-term effect, it pointed out RAO patient should be intervened as soon as possible.

This analysis has some limitations. The search identified only seven studies, with small sample sizes, which may affect the result. Three of the included studies were published in Chinese, which may cause publication bias. One study did not mention the number of patients, and thus, we used the number of affected eyes as an alternative measure [27]. Time point of vision evaluation involved in one study was from 4 months to 5 years, we used the median mentioned in the study, which was 19 months [23]. Furthermore, the search strategy was uncertain to include all of the studies, which caused potential publication bias.

## Conclusion

From a clinical perspective, oxygen therapy is a promising decongestive treatment to achieve VA improvement and favourable clinical outcomes in RAO patients. Taking all evidence into account, 100% hyperbaric oxygen and over 9 hours of treatment length is an effective clinical course.

## Supporting information

S1 Table PRISMA 2009 checklist. (DOC)

S1 File The available data file. (XLSX)
